# IL-10 Mediated Regulation of Liver Inflammation during Acute Murine Cytomegalovirus Infection

**DOI:** 10.1371/journal.pone.0042850

**Published:** 2012-08-03

**Authors:** Pamela J. Gaddi, Meredith J. Crane, Masahito Kamanaka, Richard A. Flavell, George S. Yap, Thais P. Salazar-Mather

**Affiliations:** 1 Division of Biology and Medicine, Department of Molecular Microbiology and Immunology and Warren Alpert Medical School, Brown University, Providence, Rhode Island, United States of America; 2 Department of Immunobiology, Yale University School of Medicine, New Haven, Connecticut, United States of America; 3 The Howard Hughes Medical Institute, Yale University School of Medicine, New Haven, Connecticut, United States of America; 4 Department of Medicine and Center for Immunity and Inflammation, University of Medicine and Dentistry of New Jersey-New Jersey Medical School, Newark, New Jersey, United States of America; Université Libre de Bruxelles, Belgium

## Abstract

Various cell types in both lymphoid and non-lymphoid tissues produce the anti-inflammatory cytokine interleukin (IL)-10 during murine cytomegalovirus (MCMV) infection. The functions of IL-10 in the liver during acute infection and the cells that generate this cytokine at this site have not been extensively investigated. In this study, we demonstrate that the production of IL-10 in the liver is elevated in C57BL/6 mice during late acute MCMV infection. Using IL-10 green fluorescence protein (GFP) reporter knock-in mice, designated IL-10-internal ribosomal entry site (IRES)-GFP-enhanced reporter (*tiger*), NK cells are identified as major IL-10 expressing cells in the liver after infection, along with T cells and other leukocytes. In the absence of IL-10, mice exhibit marked elevations in proinflammatory cytokines and in the numbers of mononuclear cells and lymphocytes infiltrating the liver during this infection. IL-10-deficiency also enhances liver injury without improving viral clearance from this site. Collectively, the results indicate that IL-10-producing cells in the liver provide protection from collateral injury by modulating the inflammatory response associated with MCMV infection.

## Introduction

An effective immune response against microbial pathogens is dictated by a fine equilibrium between host defense and inflammatory tissue damage. Interleukin (IL)-10, one of the most immunosuppressive cytokines, maintains this balance by dampening aspects of both innate and adaptive immunity [1]–[4]. IL-10 blocks the production of proinflammatory cytokines during the resolution period of infection and consequently reducing the immunopathology caused by these cytokines [3], [5]. IL-10 also alters the function of antigen-presenting cells such as dendritic cells and macrophages by limiting expression of MHC class II and costimulatory molecules, and the production of chemokines, thereby indirectly inhibiting T cell responses and controlling cellular accumulation [1], [4], [6], [7]. In addition to its immunosuppressive effects, IL-10 can stimulate the proliferation of B cells [1], the proliferation and activation of NK cells and cytotoxic CD8+ T cells [1], [8], as well as promote the differentiation of regulatory T cells [4].

In the context of viral infections, the presence of IL-10 may be considered either harmful or beneficial to the host. IL-10 is harmful to the host as it promotes immunosuppression and viral persistence [3], [9], [10]. In these instances, the genetic deletion of IL-10 or the blockade of its receptor augments proinflammatory and antiviral cytokine responses facilitating more efficacious viral clearance and preventing viral persistence [2], [3], [10]–[13]. While enhanced immune responses and viral eradication achieved in the absence of IL-10 may be desirable outcomes, the absence of IL-10 may promote unwanted immune mediated pathology with or without improved viral elimination depending on the site of infection [12]–[16]. Therefore, in these occasions, IL-10 may be necessary to reduce collateral damage due to inflammatory antiviral responses. These varied outcomes not only imply the central role of IL-10 in regulating the magnitude of immune responses, but also underscore the necessity for detailed studies of how IL-10 regulates immunity to infection for the benefit or detriment of the host.

Murine cytomegalovirus (MCMV) is a cytopathic herpesvirus that replicates at high levels in the spleen and liver [17], [18]. Early acute MCMV infection induces a rapid mobilization of NK cells and monocyte/macrophages into infected liver sites [19], [20]. NK cell cytotoxicity and production of antiviral cytokines such as IFN-γ and TNF-α limits MCMV infection in the liver [21], [22]. During late acute infection, between 4 and 7 days following MCMV challenge, CD8+ T cells [23] and to a lesser extent, CD4+ T cells [24], [25] accumulate and participate in antiviral responses in the liver. CD8+ T cell cytotoxicity and the release of IFN-γ and TNF-α are associated with viral elimination from this organ [17], [18], [23], [26]. Collectively, these immune responses are protective; however, they contribute to liver pathology and impaired liver function if inadequately regulated [17], [18], [22], [27], [28]. Many studies have characterized the robust proinflammatory immune responses in the liver during acute MCMV infection. By contrast, the negative regulation of these immune responses has not been extensively addressed.

Due to its immunosuppressive properties, IL-10 has been considered a factor affecting the outcome of MCMV infection and associated pathologies [28]–[33]. The suppression of macrophage and dendritic cell costimulatory properties by IL-10 has been shown to limit CD4+ T cell priming during acute MCMV infection [29]. B cell derived IL-10 diminishes CD8+ T cell activation in lymphoid organs, thereby controlling the magnitude of MCMV-specific CD8+ T cell responses [30]. Additionally, IL-10 production by CD4+ T cells hinders viral elimination in the salivary glands, consequently promoting persistence of MCMV replication within this organ [31]. Further work has described a role for IL-10 in reducing systemic IFN-γ production, CD4+ and CD8+ T cell cytokine responses and viral elimination in the spleen during MCMV infection [28]. NK cells have been implicated as sources of IL-10 in MCMV-infected perforin-deficient mice and have been suggested to restrain exaggerated CD8+ T cell responses in this model [32]. Finally, a recent study has demonstrated the impact of IL-10 in the regulation of cytokine responses and inflammation in the liver during MCMV infection, through IL-10 repletion experiments in MCMV-infected IL-10^−/−^ mice [33]. Nevertheless, it remains unclear what endogenous cellular sources of IL-10 modulate inflammatory processes in the liver in normal, wild type mice and what cellular effectors are affected by IL-10 functions in the liver during acute MCMV infection.

In this study, we investigated the kinetics and cellular sources of IL-10 in the liver during acute MCMV infection and determined that while multiple cell types produce IL-10, NK cells emerge as a prominent source of this cytokine. The absence of IL-10 during acute MCMV infection results in elevated levels of systemic and local proinflammatory cytokines and chemokines, as well as increased inflammatory cell infiltration into infected livers. These enhanced inflammatory responses were associated with increased liver pathology. However, despite augmented immune responses in infected IL-10-deficient livers, viral clearance is not improved. Together, our studies illustrate a role for IL-10-producing cells in regulating the extent of liver inflammation and injury during acute MCMV infection.

## Results

### Cellular sources of IL-10 production in liver during MCMV infection

**Figure 1:**
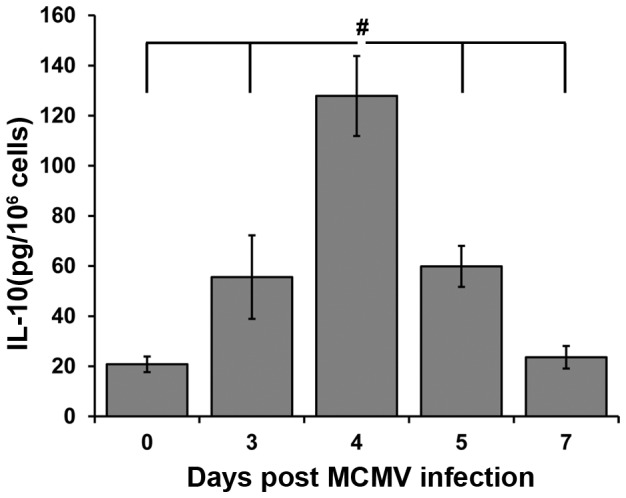
IL-10 production by liver leukocytes after MCMV infection. Liver leukocyte conditioned media were prepared from the livers of C57BL/6J mice that were uninfected (0) or infected with MCMV for 3, 4, 5, and 7 days. Concentration of IL-10 protein in liver leukocyte conditioned media was determined by standard sandwich ELISA. Results are combined data of three independent experiments (n = 5–13 mice for each time point tested). The means of IL-10 protein produced ± SE are shown. # denotes statistically significant differences between infected WT groups, where p values are ≤0.05 (one-way ANOVA, Tukey's multiple comparisons test).

To establish the time course of IL-10 production during acute MCMV infection in the liver, conditioned media were prepared from total liver leukocytes isolated from C57BL/6J (WT) mice that were uninfected or infected with MCMV for 3, 4, 5, and 7 days. IL-10 protein levels were slightly increased on day 3, but peaked significantly at day 4 after infection (Fig. 1). By days 5 and 7 post infection, the levels of IL-10 produced were reduced as compared to day 4 (Fig. 1).

**Figure 2:**
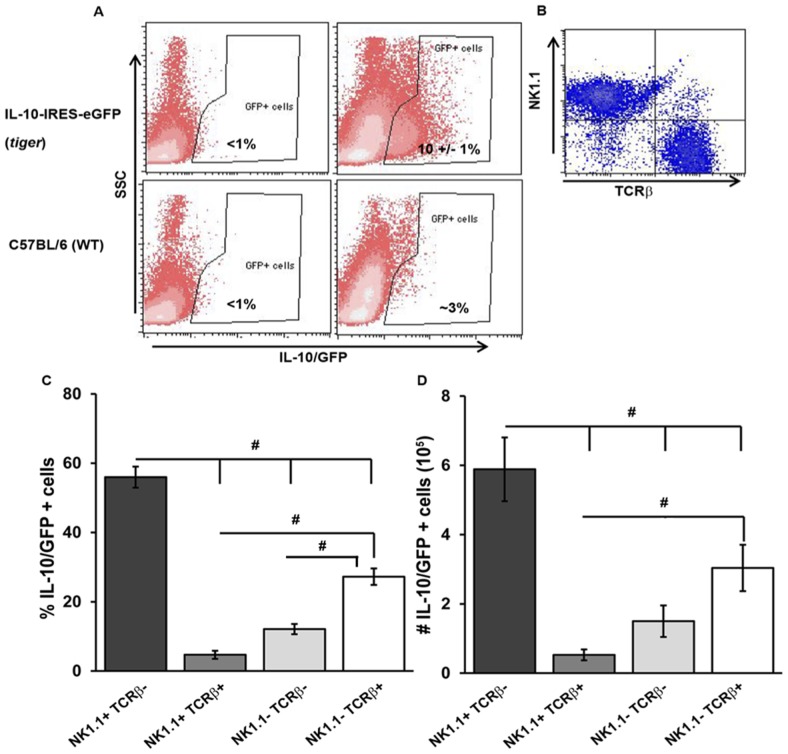
Characterization of IL-10 expressing cells in liver after MCMV infection. Liver leukocytes were prepared from IL-10-IRES-eGFP reporter (*tiger*) mice that were left uninfected (D0) or infected with MCMV for 4 days (D4). (A) The gating strategy defining IL-10/GFP positive cells by flow cytometric analysis is shown. Representative plots show the proportions of IL-10/GFP+ leukocytes after FSC/SSC gating of leukocytes and gating on IL-10/GFP+ events in relation to side scatter. D0 and D4 infected C57BL/6J (WT) mice were included to control for background fluorescence. (B) Representative plot shows NK1.1 and TCRβ expression within total IL-10/GFP+ liver leukocytes in day 4 infected *tiger* mice. (C and D) The frequency and total numbers of IL-10/GFP+ populations are shown as the means ± SE and are combined data from three independent experiments (n = 11 mice total). The statistical differences between IL-10/GFP+ groups were determined by one way ANOVA, followed by Tukey's multiple comparisons post hoc test. “#” indicates a p value of ≤0.05. The following comparisons were significant: NK1.1+ TCRβ− vs. NK1.1+ TCRβ+, NK1.1− TCRβ−, NK1.1−TCRβ+ (frequency and numbers), NK1.1+ TCRβ+ vs. NK1.1− TCRβ+ (frequency and number), and NK1.1− TCRβ− vs. NK1.1− TCRβ+ (frequency only).

Because the rise in IL-10 protein levels coincided with a period of NK cell expansion [34], [35] and T cell recruitment [23]–[26], [36] in the livers of MCMV-infected mice, we evaluated the relative contributions of NK cells and T cells to liver IL-10 expression. To identify the cells expressing IL-10 on day 4 after MCMV infection, liver leukocytes were prepared from IL-10-(IRES)-GFP-enhanced reporter (*tiger*) mice infected for 4 days. Furthermore, liver leukocytes from uninfected or day 4 MCMV-infected non-reporter C57BL/6J (WT) mice were included in the analysis to indicate background fluorescence. Analyses show that liver leukocytes from uninfected *tiger* mice marginally expressed IL-10/GFP, whereas after infection, 10%±1% of the gated leukocytes were IL-10/GFP+ (Fig. 2A). IL-10/GFP+ liver leukocytes were then characterized by their expression of NK1.1 and TCRβ and delineated as NK cells (NK1.1+ TCRβ−), T cells (NK1.1− TCRβ+), NK1.1+ T cells (NK1.1+ TCRβ+) and non-NK1.1+ non-TCRβ+ cells (NK1.1− TCRβ−) (Fig. 2B). On day 4 after infection, we found that higher frequencies of IL-10/GFP+ cells were NK1.1+ TCRβ− (NK cells), as compared to NK1.1+ TCRβ+, NK1.1− TCRβ−, and NK1.1− TCRβ+ cells (NK cells 56%±3% compared to NK1.1+ T cells: 5%±1%, and non-NK non T cells: 12%±1%, and T cells: 27%±2%) (Fig. 2C). These trends were also reflected in the absolute numbers of IL-10/GFP+ cells, with NK cells being the most numerous of the IL-10/GFP+ cells within the day 4 infected liver leukocyte population relative to other examined cell types (Fig. 2D). Further characterization of IL-10/GFP+ NK cells by flow cytometry revealed that IL-10/GFP+ NK cells (defined as NKp46+ CD3ε−) expressed known markers of maturation, DX5/CD49b, CD122, CD11b, KLRG1, and CD43 (Table S1). More than 50% of these cells were Ly49H+, CD69+, and skewed to a CD27+ CD11b+ NK cell phenotype (Table S1). Additionally, characterization of IL-10/GFP+ TCRβ+ cells demonstrated that these T cells were comprised of both CD8+ and CD4+ cells (data not shown). These results demonstrate that during acute MCMV infection, several cell types contribute IL-10 in the liver, most of which are mature and activated NK cells.

### Systemic and liver cytokine and chemokine production is amplified in the absence of IL-10 during MCMV infection

**Figure 3:**
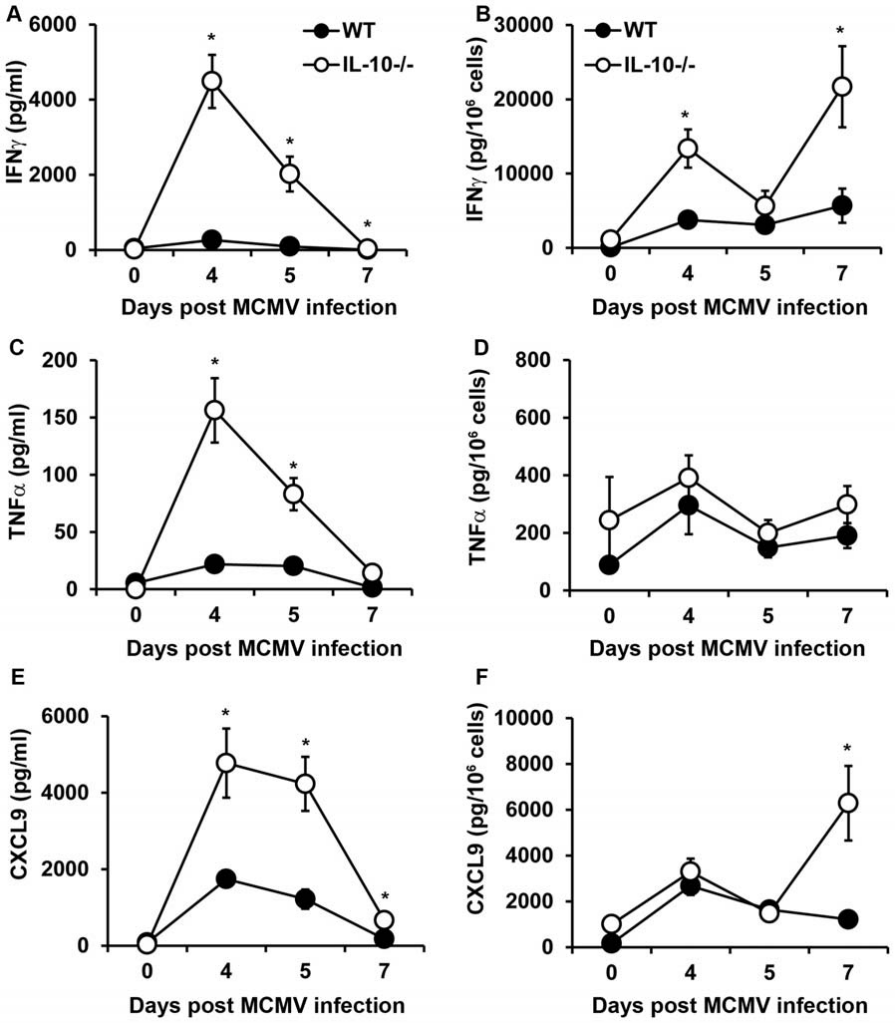
Effects of IL-10 deficiency on systemic and local proinflammatory cytokine and chemokine production after acute MCMV infection. (A, C, E) Serum samples and (B, D, F) liver leukocyte conditioned media were prepared from uninfected or MCMV-infected C57BL/6J (WT) or IL-10^−/−^ mice and tested for (A and B) IFN-γ, (C and D) TNF-α, and (E and F) CXCL9 by standard sandwich ELISA. Results are the combined data of two to four independent experiments and show the means ± SE (n = 5–18 mice per group for each time point tested). Asterisks denote statistically significant differences between WT and IL-10^−/−^ groups, where p values are ≤0.05 (Student's T test).

Since IL-10 can influence inflammation by regulating the expression of cytokines and chemokines, the effects of IL-10 deficiency on systemic and local production of key inflammatory mediators during MCMV infection were evaluated. IFN-γ and TNF-α are produced systemically and locally during this infection and limit viral replication and dissemination [17], [37], [38]. Furthermore, CXCL9/Mig, which is produced in the liver as a result of IFN-γ responses, directs the trafficking of virus-specific CD8+ T cells to this site [23]. Considering the importance of these cytokines in mediating antiviral defense, the levels of these cytokines were assessed in WT and IL-10^−/−^ mice left uninfected or infected with MCMV for 4, 5, and 7 days. Sera were collected and protein levels of IFN-γ, TNF-α, and CXCL9 were measured using ELISA (Fig. 3, A, C and E). Serum levels of all three cytokines peaked at day 4. Though levels of these cytokines declined after 4 days post infection in WT and IL-10^−/−^ mice, they remained elevated in IL-10^−/−^ mice over those in WT mice at most time points analyzed (Fig. 3, A, C and E).

Considering these observations, we next evaluated whether cytokine production by liver leukocytes during MCMV infection is similarly augmented in the absence of IL-10. ELISA was used to assess the levels of IFN-γ, TNF-α, and CXCL9 proteins in liver leukocyte conditioned media supernatants prepared from uninfected or infected WT and IL-10^−/−^ mice (Fig. 3, B, D and F). The production of IFN-γ and CXCL9 by WT and IL-10^−/−^ liver leukocytes was increased as infection progressed. The levels of IFN-γ were significantly higher on days 4 and 7 post infection in IL-10^−/−^ liver leukocytes compared to WT (Fig. 3B). Furthermore, while the kinetics of CXCL9 production were similar between WT and IL-10^−/−^ mice on days 4 and 5 post infection, CXCL9 levels produced by IL-10^−/−^ leukocytes were greater 7 days post infection as compared to levels measured in WT leukocyte conditioned media (Fig. 3F). In contrast, TNF-α levels in infected IL-10^−/−^ liver leukocytes were elevated above WT over the course of infection, though these differences were not statistically significant (Fig. 3D). Collectively, these observations indicate that IL-10 clearly suppresses the production of systemic inflammatory cytokines during MCMV infection, while the effects of IL-10 in the liver appear limited to suppression of IFN-γ and CXCL9.

### IL-10 deficiency enhances the accumulation of inflammatory effector cells in the liver during MCMV infection

**Figure 4:**
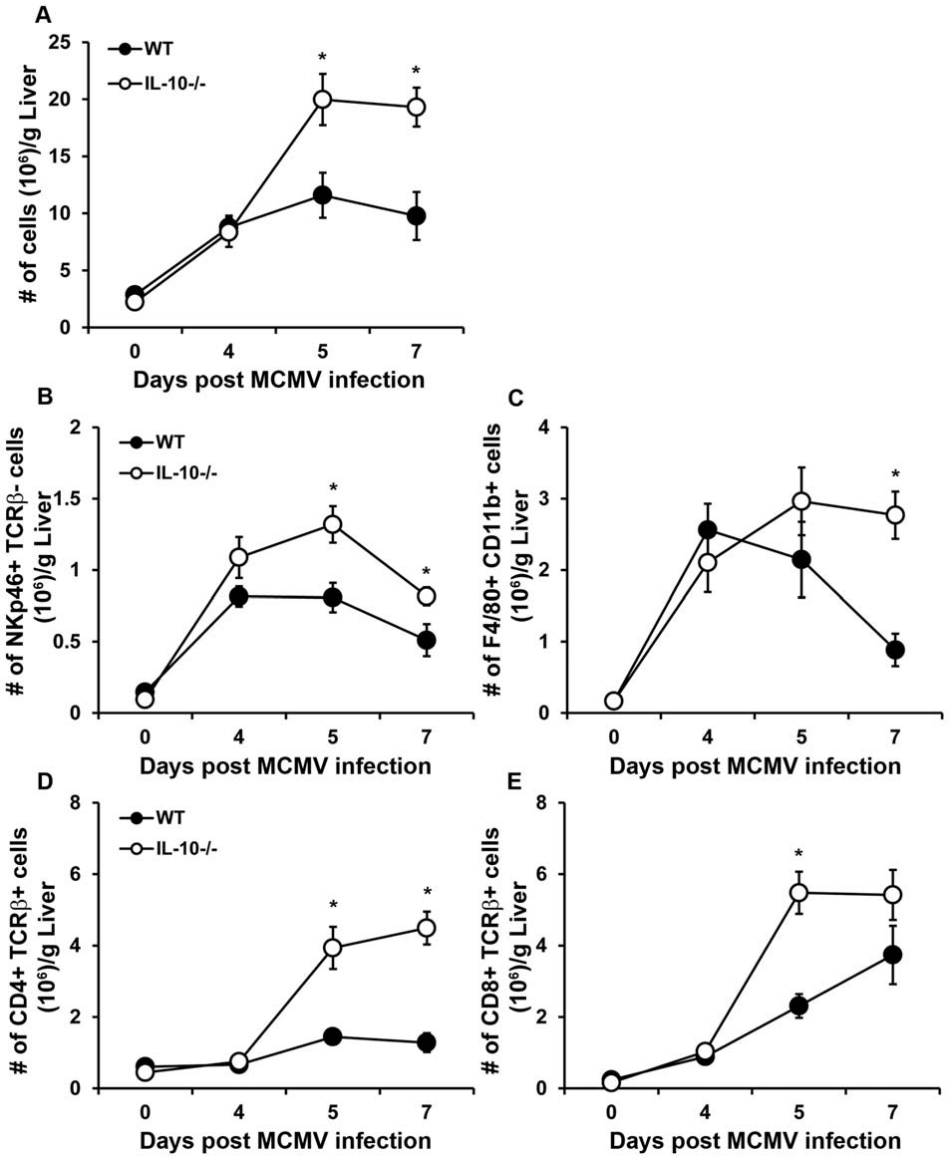
Effects of IL-10 deficiency on accumulation of inflammatory leukocytes after acute MCMV infection. Liver leukocytes were prepared from C57BL/6J (WT) or IL-10^−/−^ mice that were left uninfected or infected with MCMV for 4, 5, and 7 days. (A) The mean total liver leukocyte numbers per gram liver ± SE are shown. (B, C, D, E) Flow cytometry of effector cell populations within these total liver leukocyte populations were performed for time points indicated. Presented are the mean cell numbers per gram liver ± SE for (B) NK cells (NKp46+ TCRβ−), (C) macrophages (F4/80+ CD11b+), (D) CD4+ T cells (CD4+ TCRβ+), and (E) CD8+ T cells (CD8+ TCRβ+). (A–E) Results shown are the combined data of three independent experiments (n = 4–15 mice per group for each time point tested). Asterisks denote statistically significant differences between WT and IL-10^−/−^ groups, where p values are ≤0.05 (Student's T test).

MCMV infection in the liver results in increased infiltration of NK cells, T cells and macrophages that contribute to viral clearance through cytokine and chemokine production [20], [23], [27], [38]–[40]. The findings in Figure 3 show that IL-10 deficiency results in increased local proinflammatory cytokine production. Therefore, we hypothesized that this cytokine response could be attributed to an augmented presence of inflammatory effector leukocytes in infected IL-10 deficient livers. To assess whether IL-10 deficiency alters the infiltration of these critical immune effectors, total liver leukocyte numbers from uninfected and infected WT and IL-10^−/−^ mice were compared. As shown in Figure 4A, the numbers of infiltrating leukocytes in IL-10^−/−^ mice were increased on days 5 and 7 after infection compared to WT, implying IL-10 regulates the magnitude of leukocyte infiltration.

**Figure 5:**
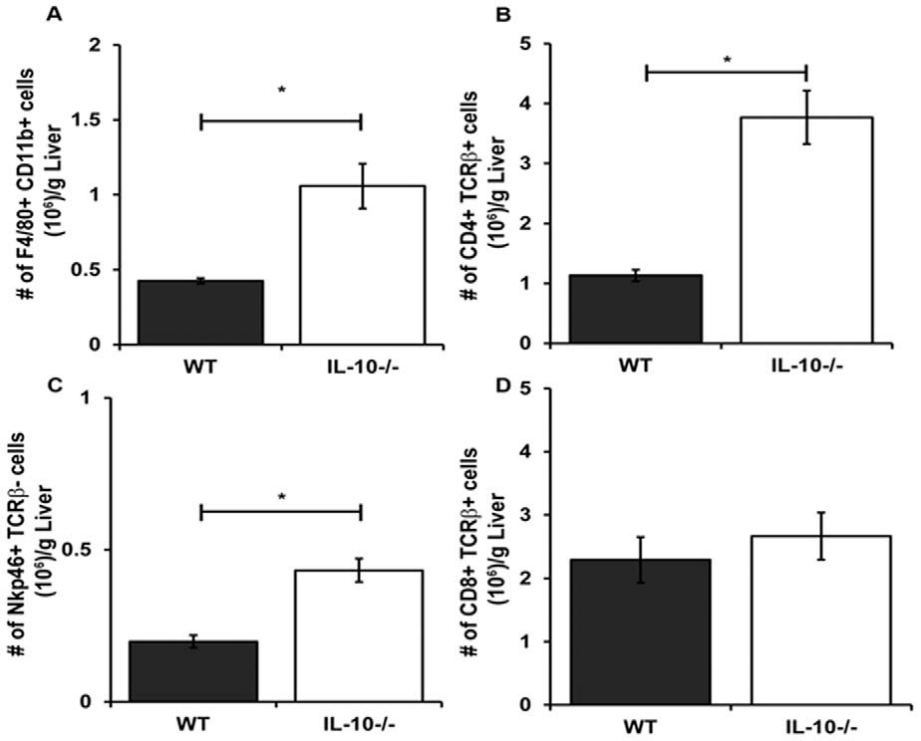
Persistence of effector cells in MCMV infected livers in the absence of IL-10. The numbers of (A) macrophages, (B) CD4+ T cells, (C) NK cells, and (D) CD8+ T cells in the livers of WT and IL-10^−/−^ mice infected for 9 days were determined by flow cytometry. The mean numbers of cells per gram liver ± SE are the combined data of four independent experiments (n = 12–13 mice per group). Asterisks denote statistically significant differences between WT and IL-10^−/−^ groups, where p values are ≤0.05 (Student's T test).

Given the increased total leukocyte infiltration in the absence of IL-10 during this infection, we investigated whether this rise in cell number was due to elevations in the numbers of a few or all of these effector cell populations. The numbers of NKp46+ TCRβ− NK cells in WT and IL-10^−/−^ livers at 4 days post infection were nearly comparable (Fig. 4B). However, NK cell numbers were higher in IL-10^−/−^ livers than in WT livers at 5 and 7 days post infection. Despite differences in the numbers of infiltrating NK cells, NK cells from IL-10^−/−^ mice exhibited similar kinetics in accumulation and contraction as those in WT mice. Like NK cells, the numbers of F4/80+ CD11b+ monocyte/macrophages were also increased on day 7 following infection (Fig. 4C) in the absence of IL-10. Furthermore, total numbers of CD4+ T cells (Fig. 4D) and CD8+ T cells (Fig. 4E, Day 5 only) were elevated in infected IL-10^−/−^ livers as compared to their WT counterparts 5 and 7 days post MCMV infection. These findings prompted further analysis of the persistence of these cells beyond day 7. By day 9 post infection the numbers of CD8+ T cells found in IL-10^−/−^ livers were comparable to WT (Fig. 5D), whereas the numbers of macrophages, CD4+ T cells, and NK cells in these same IL-10^−/−^ livers were still higher as compared to day 9 WT livers (Fig. 5, A–C). These observations suggest that IL-10 regulates the persistence of effector cells in the liver following MCMV infection.

### IL-10 deficiency enhances the accumulation of MCMV-specific CD8+ T cells

**Figure 6:**
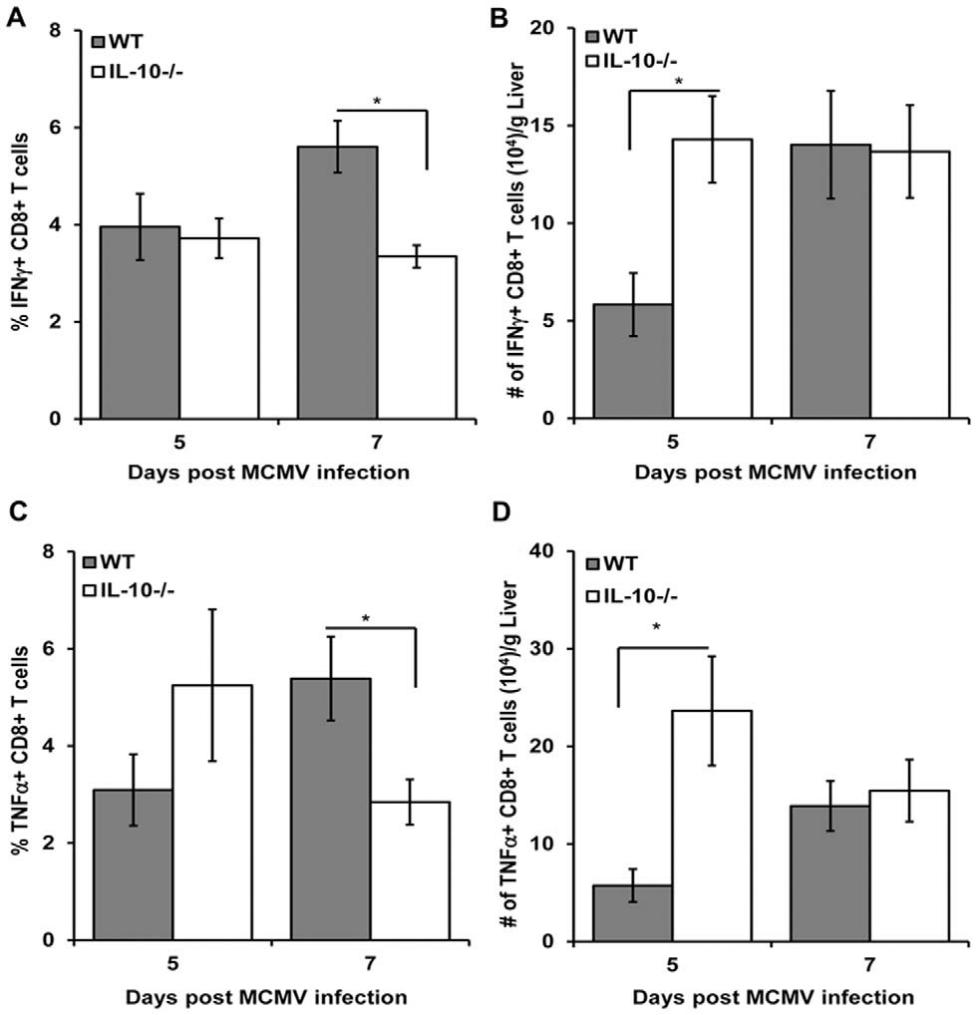
Effects of IL-10 deficiency on intracellular IFN-γ and TNF-α expression in MCMV-primed CD8+ T cells. Liver leukocytes were prepared from C57BL/6J (WT) or IL-10^−/−^ mice that were infected with MCMV for 5 and 7 days. As described in Materials and Methods, cells were stimulated with M45 viral peptide, stained for surface expression of CD8α and TCRβ, fixed, permeabilized, and stained for intracellular IFN-γ or TNF-α. Results show the frequencies total numbers of CD8+ T cells expressing (A and B) IFN-γ or (C and D) TNF-α. Data are combined from three independent experiments (n = 9–10 mice per group for each time point tested) and show the means ± SE. Asterisks denote statistically significant differences between WT and IL-10^−/−^ groups, where p values are ≤0.05 (Student's T test).

Virus-specific CD8+ T cells are recruited to the liver within 4 days of infection and control viral replication through release of cytotoxic molecules and production of cytokines such as IFN-γ and TNF-α [18], [21]–[23], [26]. Given the augmented accumulation of CD8+ T cells in infected IL-10^−/−^ livers noted above, we evaluated whether IL-10 deficiency affected the infiltration of cytokine-producing CD8+ T cells activated by an immunodominant epitope of MCMV, an H-2D^b^ viral peptide derived from the MCMV M45 protein [41]–[43]. The frequencies and total numbers of antigen-specific IFN-γ and TNF-α producing CD8+ T cells were determined by intracellular staining for these cytokines after restimulation with this viral peptide. The results shows that the frequency of IFN-γ+ (Fig. 6A) and TNF-α+ (Fig. 6C) CD8+ T cells on day 5 post infection were comparable between WT and IL-10^−/−^ mice. Interestingly, IL-10^−/−^ mice exhibited decreased frequencies of both IFN-γ+ (Fig. 6A) and TNF-α+ (Fig. 6C) CD8+ T cells on day 7 post infection. The total numbers of IFN-γ+ (Fig. 6B) and TNF-α+ (Fig. 6D) CD8+ T cells in day 5 infected IL-10^−/−^ livers were increased as compared to WT mice. Despite these differences on day 5, the numbers of both IFN-γ+ and TNF-α+ CD8+ T cells in IL-10^−/−^ livers from mice infected for 7 days were comparable to those numbers in WT (Fig. 6, B and D). Taken together, these observations indicate that IL-10 limits the number of activated virus-specific CD8+ T cells in the liver.

### IL-10 deficiency increases hepatic immunopathology during MCMV infection

Our previous observations demonstrated that IL-10 deficiency leads to inflated cellular and cytokine responses in the liver during this infection. We next sought to determine whether such inflammatory events exacerbated liver damage in infected IL-10^−/−^ mice. The formation of inflammatory clusters in the liver after MCMV infection has been correlated with the onset of damage as well as the establishment of antiviral responses at this site [20], [27], [44]. To dissect the consequences of IL-10 deficiency on liver inflammation after infection, we first quantitated the number of inflammatory foci in livers using hematoxylin and eosin (H&E) stained liver sections taken from uninfected and MCMV-infected WT and IL-10^−/−^ mice. We noted no differences in the numbers of inflammatory clusters counted in WT and IL-10^−/−^ livers infected for 4 days (Table 1). However, by days 5 and 7 post infection, there were increased numbers of inflammatory foci observed in IL-10^−/−^ livers as compared to WT. By day 9 post infection, the number of foci in IL-10^−/−^ livers was nearly comparable to WT. Furthermore, the number of nucleated cells within each focus was greater in IL-10^−/−^ livers than in WT livers infected for 5 and 7 days (Table 1), thus, corroborating our previous observations of increased leukocyte infiltration (Fig. 4A).

**Table 1:** Quantitation of inflammatory foci in MCMV infected C57BL/6J (WT) and IL-10^−/−^ livers.

Days post MCMV infection	No. of foci counted a		No. of nucleated cells per focus b	
	WT	IL-10^−/−^	WT	IL-10^−/−^
0	-	-	-	-
4	9±2	12±1	-	-
5	11±2	23±1^a^	41±4	>60^c^
7	3±0.4	9±1^a^	26±2	38±4^b^
9	2±1	4±1	-	-

a Number of foci were quantified in a 400 µm^2^ total liver area from 8 fields of view (FOV), each FOV with a square area of 50um^2^ at a magnification of 200. Data are from at least three independent experiments with n = 4–11 mice per group and show the mean numbers of foci per 400 µm^2^ total liver area ± SE, where p≤0.05 (Student's T test) was considered significant.

b Number of nucleated cells per inflammatory focus was determined by counting individual cells within 20 random foci, under a magnification of 400. Data were combined from at least three experiments (n = 9–11 mice per group for each time point tested) and indicate the mean number of cells per focus ± SE where p≤0.05 (Student's T test) was considered significant.

c Foci containing cell numbers of >60 result in less defined foci and results do not indicate SE.

**Figure 7:**
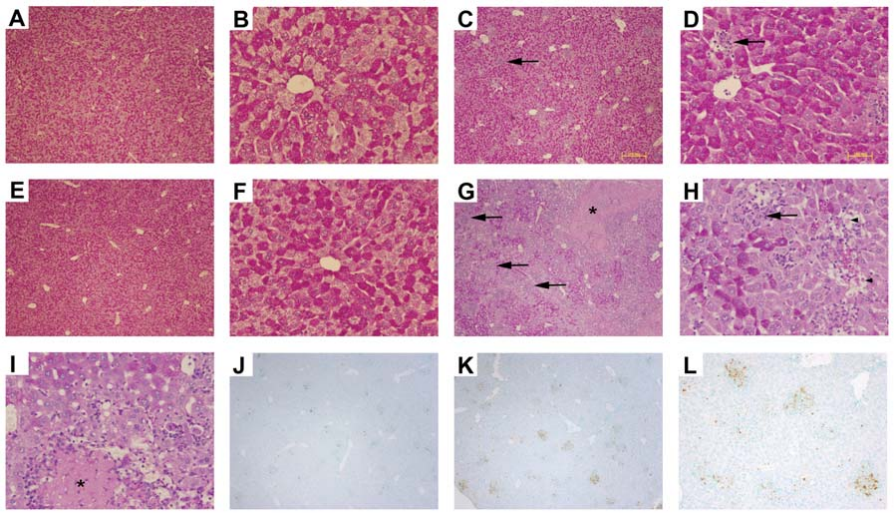
Liver pathology during acute MCMV infection in the absence of IL-10. Livers from (A, B, C, D) C57BL/6J (WT) and (E, F, G, H) IL-10^−/−^ mice that were (A, B, E, F) uninfected or (C, D, G, H, I, J, K, L) infected with MCMV for 5 days were harvested, paraffin embedded, and sectioned for PAS staining. Inflammatory foci (arrows, C, D, G & H), necrosis (asterisks, G and I), councilman bodies (black arrowheads, H), are marked. TUNEL positive cell clusters are shown in (J) Day 5 WT and (K and L) IL-10^−/−^ livers. A, C, E, G, J & K were visualized at ×4 magnification. B, D, F, H, & I at ×20 magnification. L was visualized at ×10 magnification.

Further evaluation of liver pathology and damage in the absence of IL-10 during this infection was performed using periodic acid Schiff stained (PAS) liver sections taken from uninfected and infected WT and IL-10^−/−^ mice. PAS staining has been utilized as an indicator of liver function or glycogen storage, whereby reductions of staining denote impaired liver function. Using this approach, we revealed no distinct differences in PAS staining between the livers of uninfected WT (Fig. 7, A and B) and IL-10^−/−^ (Fig. 7, E and F) mice. On day 4 post infection, both WT and IL-10^−/−^ livers had comparable reductions in PAS staining and an increased presence of inflammatory foci (data not shown). On day 5 after infection, WT mice exhibited reduced liver PAS staining (Fig. 7, C and D), though this reduction in positive PAS staining was markedly more apparent in IL-10^−/−^ mice (Fig. 7, G and H). In contrast, the extent of positive PAS staining in liver sections from day 7 infected IL-10^−/−^ and WT mice were comparable (data not shown), indicating recovery of glycogen synthesis. Concurrent with recent reports [33], day 5 infected IL-10^−/−^ livers revealed more focal necrosis (Fig. 7, G and I) and inflammatory foci (Fig. 7 G and H) as compared to WT livers. Furthermore, day 5 infected IL-10^−/−^ livers had more councilman bodies (apoptotic hepatocytes) (Fig. 7H) and demonstrated increased TUNEL staining (Fig. 7, K and L) as compared to day 5 infected WT livers (Fig. 7J).

The extent of liver damage in infected IL-10^−/−^ mice was also assessed by measurement of serum alanine aminotransferase levels (ALT) in uninfected and infected WT and IL-10^−/−^ mice. WT and IL-10^−/−^ mice exhibited elevated serum ALT on day 4 and 5 after infection, with serum values in the IL-10^−/−^ mice higher than WT on day 5 post infection, though these differences did not attain statistical significance (Table 2). On day 7 after infection, serum ALT levels had declined in both WT and IL-10^−/−^ mice, although the levels in IL-10^−/−^ mice remained elevated compared to WT levels (Table 2). Finally, viral titers of infected WT and IL-10^−/−^ livers were examined to determine whether the inflated immune responses and resultant immunopathology in infected IL-10^−/−^ livers coincided with improved viral clearance. Despite the increased immune responses and pathology during infection in the absence of IL-10, viral titers in the IL-10^−/−^ livers remained comparable to those in WT livers at all time points analyzed (Table 3). Taken together, these results indicate that IL-10 minimizes immunopathology during acute MCMV infection without affecting viral clearance in the liver.

**Table 2:** Serum alanine aminotransferase (ALT) levels of uninfected and MCMV infected C57BL/6 (WT) and IL-10^−/−^ mice.

Days post infection	Serum ALT (U/L) a	
	WT	IL-10^−/−^
0	65.1±11.9	44.9±3.0
4	312±20.8	281.2±21.1
5	233.3±33.3	358.8±69.8
7	52.1±2.9	85.5±6.5^b^

a ALT values expressed in units/liter (U/L) are the cumulative data of four independent experiments and are presented as the means ± SE (n = 7–16 mice per group for each time point tested).

b p≤0.05 when compared to WT mice at day 7 after infection (Student's T test).

**Table 3:** Viral titer measurements of MCMV infected C57BL/6 (WT) and IL-10^−/−^ livers.

Days post infection	Viral titer log PFU/liver g a	
	WT	IL-10^−/−^
0	0	0
4	3.33±0.09	3.41±0.17
5	2.84±0.07	2.40±0.36
7	1.67±0.54	2.00±0.41

a Mice were uninfected or infected with MCMV as indicated. Viral titers were measured by plaque assay as described in Materials and Methods and expressed as log plaque forming units (PFU) per liver gram. The limit of detection for this assay was 2 log PFU/g liver. Results shown are the means ± SE of cumulative data from two independent experiments (n = 4–9 mice per group for each time point tested).

## Discussion

This study evaluated the production and the function of IL-10 in the liver during acute MCMV infection. Our results show IL-10 is produced in the liver at a key time point before the accumulation of T cells and after the early effector activities of NK cells [23], [24], [34], [35], [38]. Furthermore, they highlight NK cells as major sources of IL-10, though other cells, such as T cells and mononuclear cells also contribute IL-10. The absence of IL-10 during acute infection results in inflated proinflammatory cytokine and chemokine production and accumulation of inflammatory cells in infected livers. These unrestrained inflammatory responses promote the development of liver injury, but do not enhance viral elimination at this site.

We identified mature, activated NK cells as prominent IL-10 expressing cells during acute MCMV infection. These results concur with recent studies demonstrating the presence of IL-10 producing NK cells during various microbial infections [32], [45], [46]. During MCMV infection, IL-10 production by NK cells has been implicated in modulating over-exaggerated CD8+ T cell responses [32]. Our studies suggest that through the production of IL-10, NK cells may participate in dampening downstream effector responses and subsequent immunopathology in the liver during acute MCMV infection.

Notably, although NK cells appear to be predominant IL-10 expressers, T cell and non-NK, non T cell populations also comprise the IL-10+ leukocyte population in the liver after MCMV infection. Macrophages and dendritic cells [29], B cells [30] and CD4+ T cells [31], [47] have been shown to produce IL-10 during this infection, though their contributions to IL-10 production in acutely infected livers remain to be examined. Virus-specific CD8+ T cells have been identified as important IL-10 producers during infections of influenza A [48], respiratory syncytial virus [15] and coronavirus [49]. It is possible that MCMV-specific, effector CD8+ T cells and CD4+ T cells contribute IL-10 in the liver during this infection, although further characterization of IL-10+ T cells at this site is necessary as regulatory subsets of CD4+ T cells [50] and CD8+ T cells [51], [52] have been shown to also generate IL-10 in various contexts. Finally, our study cannot rule out the contributions of specific NK1.1− TCRβ− cells, which may include macrophages and dendritic cells, to IL-10 mediated regulation in the liver. Peritoneal and splenic macrophages and dendritic cells produce IL-10 after MCMV infection [29] and it is known that monocyte/macrophages accumulate in the liver during infection [18], [38], [39]. Therefore, further studies are necessary to delineate IL-10+ cells within the NK1.1− TCRβ− population and the TCRβ+ population, as these may also contribute to the regulation of inflammation in the liver during infection.

The immune response to MCMV infection involves the mobilization and activation of NK cells, T cells, and monocyte/macrophages, which are sources of proinflammatory cytokines and chemokines [17], [18], [23], [38], [44], [53]. Consistent with previous observations [28], [33], we found that in the absence of IL-10 there is augmented cellular infiltration of livers and increased levels of IFN-γ and CXCL9. Though TNF-α production is involved in the cytokine response during MCMV infection, we note that TNF-α protein levels, though increased systemically in the absence of IL-10, remained unchanged in the liver during acute MCMV infection, corroborating with reported transcript data [33]. Elevated levels of IFN-γ and CXCL9 may partly account for the increased CD8+ T cell accumulation in MCMV-infected IL-10^−/−^ livers as studies have associated liver production of IFN-γ and CXCL9 with recruitment of MCMV-specific CD8+ T cells to the liver during MCMV infection [23], [38]. Additionally, the increased cellular accumulation in MCMV-infected IL-10^−/−^ livers may also reflect the broad immunosuppressive impact of IL-10 on cellular activation and proliferation [1]–[5]. IL-10 dampens macrophage proliferation and T cell priming [1], [4], [6], [7], and during acute MCMV infection, IL-10 limits CD8+ T cell numbers in lymphoid organs [28], [30], [32], thereby, perhaps restricting the numbers of cells recruited to the liver.

Immune responses to MCMV precipitate the development of liver pathology during late acute MCMV infection [17], [22], [27], [33], and our studies and others [33] suggest that in the absence of IL-10, liver damage is enhanced. Although the cells and cytokines that mediate liver damage in IL-10^−/−^ mice have yet to be clearly identified, it has been established that overproduction of IFN-γ and TNF-α can promote the development of severe hepatitis during acute MCMV infection [18], [22], [27]. Moreover, excess production of these cytokines by activated monocytes/macrophages, NK cells, CD4+ and CD8+ T cells have been associated with MCMV-induced liver disease in immunodeficient mice [22]. It is probable that increased cellular accumulation and the elevated production of inflammatory mediators such as IFNγ and CXCL9 as well as others not evaluated by this study accounts for the extent of liver disease in observed IL-10 deficient livers. Our findings support the conclusions of recent studies in MCMV infection [33] and other viral infections [16], [48] that demonstrate IL-10 as a key cytokine in limiting immune mediated pathology during viral infection.

Despite increased effector cell infiltration and cytokine production in virally infected IL-10 deficient livers, we did not detect any significant differences in viral titers between WT and IL-10^−/−^ livers, concurring with a recent report [33]. These findings contrasted other studies, which demonstrated IL-10 suppresses MCMV elimination in the spleen [28] and salivary glands [31]. These differences may reflect compartmental differences in viral elimination and in regulation of antiviral immune responses. Lack of improved clearance in IL-10 deficient livers could be attributed to the activity of other immunoregulatory mechanisms such as programmed death-1(PD-1)-PD-L1 (PD ligand-1) or Tim-3-galectin-9, which are known to negatively regulate T cell responses. Interestingly, multiple cell types in the liver express the ligands to these molecules [54]–[57], and PD-1 and Tim-3 have been shown to be upregulated on CD8+ T cells during both acute [58]–[61] and chronic persistent viral infections [10], [61]–[64]. Further work exploring the roles of inhibitory receptor-ligand interactions during acute MCMV infection in the liver in the absence of IL-10 may elucidate mechanisms underlying compartmental differences in viral elimination.

In conclusion, the current studies demonstrate a role for IL-10 in regulating inflammatory responses involved in the elimination of MCMV infection in the liver. NK cells are identified as prominent expressers of IL-10, although other cell populations also contribute to IL-10 in the liver during this infection. The absence of IL-10 mediated suppression during MCMV infection results in amplified cellular and cytokine responses that ultimately result in liver injury. In spite of a robust immune response in infected IL-10-deficient livers, viral clearance is not improved. Therefore, our results highlight the role for IL-10 and cellular sources of IL-10 in the protection of the liver from damage due to infection induced inflammatory responses.

## Materials and Methods

### Ethics statement

This study was carried out in strict accordance with the recommendations in the Guide for the Care and Use of Laboratory Animals of the National Institutes of Health. All animal work was approved by the Brown University Institutional Animal Care and Use Committee (Protocol number: 0903035).

### Mice

Pathogen-free C57BL/6J and IL-10^−/−^ mice were obtained from the Jackson Laboratory (Bar Harbor, ME, USA). B6-IL-10-internal ribosome entry site (IRES) green fluorescence protein (GFP) knockin (IL-10-IRES-GFP) reporter *tiger* mice were generated as described in [65] and bred in pathogen-free mouse facilities at Brown University. All mice were maintained in pathogen-free mouse facilities at Brown University. Age- (6–8 week old) and sex-matched mice were used in all experiments.

### Virus and viral titer determination

Infections were initiated on day 0 by intraperitoneal (i.p) injection of 5×10^4^ PFU of salivary gland-passaged MCMV Smith strain prepared as previously described [40], [66]. *In vivo* responses were examined at indicated times post infection. Viral titer quantification was done as described (66). Briefly, duplicate samples of serially diluted homogenates were plated on bone marrow stromal cell (M2-10B4) (American Type Culture Collection, Manassas, VA) monolayers for one hour at 37°C, 5% CO_2_. After 1 h incubation, inocula were removed from monolayers. Monolayers were overlaid with 1× DMEM/0.5% low melt agarose solution and further incubated for 7 days. Infected monolayers were fixed with 10% buffered formalin and stained with crystal violet to allow for visualization of plaques. Plaques were counted to calculate viral titers as previously described [23], [39], [66].

### Preparation of leukocytes, serum, and conditioned media

Livers harvested from infected WT and IL-10^−/−^ mice were weighed and then processed to isolate single-cell liver suspensions as previously described [19], [23], [38]. For generation of leukocyte conditioned media, isolated leukocytes were plated without additional stimulation in round-bottom microtiter plates at 10^6^ cells/well in RPMI 1640 (Invitrogen), supplemented with 10% heat-inactivated fetal calf serum (Atlanta Biologicals). After 24 h of incubation at 37°C, cell free supernatants were collected and stored at −20°C until used in cytokine analyses. Blood serum was collected following centrifugation of whole blood collected into heparin-containing tubes and stored at −80°C until further use in cytokine analyses or alanine aminotransferase assays.

### Flow cytometric analyses

Liver leukocytes were incubated with anti-CD16/32 antibody (clone 2.4G2) (BD Biosciences/Pharmingen, San Diego, CA, USA) prior to staining to block non-specific binding. To identify inflammatory leukocytes, the following antibodies were used in surface staining: PE-conjugated anti-F4/80 (AbD Serotec, Raleigh, NC, USA), APC-conjugated anti-CD11b, PeCy7 or PerCP-Cy5.5-conjugated anti-CD8α, FITC- or APC-conjugated anti-TCRβ, PerCP-conjugated anti-CD4, PeCY7 or PE-conjugated anti-NK1.1, and eFluor-710- or PerCP-Cy5-conjugated anti-NKp46. For phenotypic characterization of IL-10/GFP+ NK cells, in addition to NK specific antibodies for NK1.1 and NKp46 listed above, Alexa 647 conjugated anti-Ly49H, APC-conjugated anti-DX5/CD49b, anti-CD43, anti-KLRG1, and -anti-CD69, Pacific Blue-conjugated anti-CD122, Pacific Blue- conjugated anti-CD11b, and PECy7-conjugated anti-CD27, and PE-conjugated anti-CD3ε antibodies were utilized. All antibodies, except for anti-F4/80 (AbD Serotec), were obtained from BD Biosciences or eBioscience (San Diego, CA, USA), unless otherwise noted. Isotype control antibodies were used to set analysis gates. Where indicated, GFP expression in leukocytes from *tiger* mice was analyzed in the FITC/FL1 channel. Approximately 70,000–100,000 events per sample were acquired using a FACSCalibur or FACSAria and data was analyzed using BD CellQuest software. Where indicated, positive GFP gates were set using C57BL6J negative controls. For phenotypic characterization of IL-10/GFP+ NK cells, a lymphoid gate was used in analysis and NK cells were defined as NKp46+ CD3ε−.

### Peptide-specific in vitro stimulations and intracellular staining

To evaluate the cytokine production by MCMV specific CD8+ T cells, total liver leukocytes from uninfected or MCMV-infected mice were cultured in complete media and 100 ng/ml of the immunodominant peptide from the M45 viral protein [23], [41]–[43] for a total of 5 h with brefeldin A (eBioscience), which was added within the last 3 h of this incubation period. After 5 h of incubation, leukocytes were stained with PE-Cy7- or PerCP-conjugated anti-CD8α, fixed, permeabilized (Cytofix/Cytoperm; BD Biosciences), and incubated with PE-conjugated anti-mouse IFN-γ (clone XMG1.2), APC-conjugated anti-TNF-α (clone MP6-XT22), or isotype control IgG1 (BD Biosciences). Isotype control antibodies were used to correct for background fluorescence and set analysis gates. Cells were acquired and analyzed using FACSCalibur and CellQuest-Pro software.

### Cytokine analysis

Serum and leukocyte-conditioned media were tested for IL-10, IFN-γ, TNF-α and CXCL9 using DuoSets (R&D Systems, Minneapolis, MN). The limits of detection were 15–32 pg/10^6^ cells.

### Histology and hepatic enzyme analyses

Liver lobes were fixed in 10% buffered formalin and paraffin-embedded for sectioning. Tissue sections of 5 µm in thickness were cut and stained with hematoxylin and eosin (H&E) and periodic acid Schiff (PAS) stain for microscopic evaluations. For in situ detection of apoptotic cells, TdT-mediated dUTP-biotin nick end labeling (TUNEL) assay was used as described by manufacturer (Millipore, Temecula, CA, USA). Inflammatory foci, which are defined as discrete clusters of 6–60 nucleated cells [27] were quantified by analysis of eight 50 µm^2^ fields of view on H&E stained liver sections under a magnification of 200. The number of nucleated cells per inflammatory focus was determined by counting cells within 20 random foci per representative H&E stained liver section at a magnification of 400. Images were photographed with a DP70 digital camera and software (Optical Analysis Corporation, Nashua, NH, USA). Serum ALT levels were measured by Marshfield Labs (Marshfield, WI, USA).

### Statistical analyses

Statistical differences in experimental results were determined using Graphpad Prism version 5 and Excel. Statistical differences for the frequencies and numbers of IL-10/GFP+ groups and in IL-10 levels measured in the conditioned media of multiple WT infection groups were assessed by one way analysis of variance (ANOVA) test, accompanied by Tukey's multiple comparisons post hoc test. For these multiple comparisons tests, p values of ≤0.05 were considered significant and denoted with a “#”. Furthermore, differences between WT and IL-10^−/−^ groups in cell number, cytokine levels for serum and liver leukocyte conditioned media, foci number, and serum ALT levels were determined by Student's T tests, where p values of ≤0.05 were significant. For comparisons made between phenotyped IL-10/GFP+ NK cells versus IL-10/GFP- NK cells, differences were also evaluated using Student's T tests, where p values of ≤0.05 were significant.

## Supporting Information

Table S1 **Expression of maturation and activation markers on IL-10/GFP+ NK cells from day 4 infected livers.**
(DOCX)
