# Supplementary material for: IL-10 Mediated Regulation of Liver Inflammation during Acute Murine Cytomegalovirus Infection
Source: PLoS One. 2012 Aug 3;7(8):e42850. doi: 10.1371/journal.pone.0042850 (PMC3411849; doi:10.1371/journal.pone.0042850)
Supplement: Table S1 — Expression of maturation and activation markers on IL-10/GFP+ NK cells from day 4 infected livers. (DOCX) [file pone.0042850.s001.docx]

Table S1. Expression of maturation and activation markers on IL-10/GFP+ NK cells from day 4 infected livers.

| Marker expressed | % of IL-10/GFP+ NK cells ± SE^a^ | % of IL-10/GFP- NK  cells ± SE^a^ |
| --- | --- | --- |
| NK1.1 | 85.8 ± 1.5 | 84.9 ± 1.4 |
| DX5/CD49b | 91.9 ± 2.0 | 89.2 ± 0.4 |
| CD122 | 97.8 ± 0.5^b^ | 92.2 ± 0.5 |
| CD11b | 92.0 ± 1.3 | 88.9 ± 2.0 |
| CD43 | 82 ± 4.1 | 81.7 ± 3.4 |
| KLRG1 | 64.9 ± 1.4 | 63.6 ± 0.9 |
| Ly49H | 62.5 ± 2.2 | 59.5 ± 2.8 |
| CD69 | 92.1± 0.7^b^ | 78.1 ± 1.7 |
| CD27+ CD11b+ | 75.5 ± 1.6^b^ | 64.6 ± 1.5 |
| CD27- CD11b+ | 16.3 ± 1.2 | 24.3 ± 2.2^b^ |
| CD27+ CD11b- | 7.6 ± 1.2 | 8.1 ± 1.6 |
| CD27- CD11b- | 1.5 ± 0.4 | 2.9 ± 0.3^b^ |

^a^ Data summarized are the mean frequencies of marker positive cells ± SE within IL-10/GFP+ NK cells and IL-10/GFP- NK cells (where NK cells are defined as NKp46+ CD3ε-) from day 4 infected IL-10/GFP+ liver leukocytes. Results shown are the combined data from two independent experiments (n= 5-13 mice per marker tested).

^b^ Denotes significant differences between the means of IL-10/GFP+ NK cells and IL-10/GFP- NK cells, where p ≤ 0.05 (Student’s T test).
